# Differential roles of TGIF family genes in mammalian reproduction

**DOI:** 10.1186/1471-213X-11-58

**Published:** 2011-09-29

**Authors:** Yanqiu Hu, Hongshi Yu, Geoff Shaw, Marilyn B Renfree, Andrew J Pask

**Affiliations:** 1 ARC Centre of Excellence for Kangaroo Genomics, Department of Zoology, The University of Melbourne, VIC, 3010, Australia; 2 Department of Molecular and Cellular Biology, The University of Connecticut, Storrs, CT 06269, USA

## Abstract

**Background:**

TG-interacting factors (TGIFs) belong to a family of TALE-homeodomain proteins including TGIF1, TGIF2 and TGIFLX/Y in human. Both TGIF1 and TGIF2 act as transcription factors repressing TGF-β signalling. Human *TGIFLX *and its orthologue, *Tex1 *in the mouse, are X-linked genes that are only expressed in the adult testis. *TGIF2 *arose from *TGIF1 *by duplication, whereas *TGIFLX *arose by retrotransposition to the X-chromosome. These genes have not been characterised in any non-eutherian mammals. We therefore studied the TGIF family in the tammar wallaby (a marsupial mammal) to investigate their roles in reproduction and how and when these genes may have evolved their functions and chromosomal locations.

**Results:**

Both *TGIF1 *and *TGIF2 *were present in the tammar genome on autosomes but *TGIFLX *was absent. Tammar *TGIF1 *shared a similar expression pattern during embryogenesis, sexual differentiation and in adult tissues to that of *TGIF1 *in eutherian mammals, suggesting it has been functionally conserved. Tammar *TGIF2 *was ubiquitously expressed throughout early development as in the human and mouse, but in the adult, it was expressed only in the gonads and spleen, more like the expression pattern of human *TGIFLX *and mouse *Tex1*. Tammar *TGIF2 *mRNA was specifically detected in round and elongated spermatids. There was no mRNA detected in mature spermatozoa. TGIF2 protein was specifically located in the cytoplasm of spermatids, and in the residual body and the mid-piece of the mature sperm tail. These data suggest that tammar *TGIF2 *may participate in spermiogenesis, like *TGIFLX *does in eutherians. *TGIF2 *was detected for the first time in the ovary with mRNA produced in the granulosa and theca cells, suggesting it may also play a role in folliculogenesis.

**Conclusions:**

The restricted and very similar expression of tammar *TGIF2 *to X-linked paralogues in eutherians suggests that the evolution of *TGIF1*, *TGIF2 *and *TGIFLX *in eutherians was accompanied by a change from ubiquitous to tissue-specific expression. The distribution and localization of TGIF2 in tammar adult gonads suggest that there has been an ultra-conserved function for the TGIF family in fertility and that *TGIF2 *already functioned in spermatogenesis and potentially folliculogenesis long before its retrotransposition to the X-chromosome of eutherian mammals. These results also provide further evidence that the eutherian X-chromosome has actively recruited sex and reproductive-related genes during mammalian evolution.

## Background

Homeobox genes are characterized by a conserved 180 base pair motif encoding a homeodomain with three structurally conserved helices [1]. The TALE superfamily is distinguished by a three-amino-acid loop extension (TALE) between helix 1 and helix 2 within the homeodomain [2,3]. The third helix is highly conserved playing the major role in DNA binding site recognition [3]. TALE homeodomain proteins are critical transcription factors for embryonic and early development [4,5]. *TGIF *(named after transforming growth factor-β-induced factor or 5'-TG-3' interacting factor) genes are members of TALE superfamily, containing *TGIF1*, *TGIF2*, *TGIFLX *and *TGIFLY *[2].

*TGIF1 *orthologues share a high similarity inside the homeodomain between distantly related vertebrates [6]. TGIF1 is a Smad transcriptional co-repressor that negatively regulates TGF-β-activated gene expression in vertebrates [6,7]. In *Drosophila*, TGIF1 is a transcriptional activator interacting with Mad and Smad2 [6]. In humans, *TGIF1 *is highly expressed in the placenta and other adult tissues such as liver, kidney and gonads [3]. Mutations in *TGIF1 *can cause holoprosencephaly (HPE), a severe disease affecting forebrain and craniofacial development, associated with mental retardation [8]. Two isoforms of *Tgif1 *were identified by RT-PCR from alternative splicing events that are specific to the mouse [9]. Both alternative splice forms are functional as transcriptional repressors [9]. Mouse *Tgif1 *mRNA is initially detected at E9.5 with the highest expression in the forebrain, the branchial arches, the otic pit, and the limb buds but not in the heart [10]. Subsequently the expression is maintained at a higher level from then on in the forebrain but declines throughout the whole embryo by E14.5 [10,11]. However, *Tgif1 *knockout mice do not have any abnormal phenotypes [10], possibly due to a functional redundancy with *Tgif2*, since both occupy similar spatial and temporal expression domains during embryogenesis [10] and both are co-repressors for TGF-β receptor activated Smads by interacting with histone deacetylases (HDACs) [4].

TGIF2 shares similar DNA binding homeodomains to TGIF1, suggesting both proteins are likely to bind the same DNA sequence [4]. Human *TGIF2 *has 3 exons which have the highest homology with *TGIF1*. There are two alternative splicing forms of *Tgif2 *genes one of which contains a retained intron within the second coding exon that occurs only in mice [9]. Both forms of *Tgif2 *mRNA transcripts are present and functional as transcriptional repressors in adult and embryonic tissues in mice and have similar expression patterns [9]. *TGIF2 *is ubiquitously expressed in human tissues, with particularly high expression in the heart, kidney and testis, but the transcript is almost undetectable in the brain and prostate [12]. In the mouse, *TGIF2 *transcripts are highly expressed in the nervous system at E12.5 and E15.5, indicating this gene has a wide but well controlled expression pattern during early embryo development [10,13]. Both *Tgif1 *and *Tgif2 *are required for gastrulation in mice and act to limit *Nodal *signalling and L-R axis specification [5].

In addition to the autosomal TGIFs described above, orthologues exist on the eutherian mammal × and Y-chromosomes, *TGIFLX *(transforming growth factor-β-induced factor 2-like, X-linked) and *TGIFLY *(transforming growth factor-β-induced factor 2-like, Y-linked). TGIFLY lacks the specific C-terminal residues shared by TGIFLX, TGIF1 and TGIF2 [14]. The *TGIFLX *gene originated from autosomal *TGIF2 *by retrotransposition [14], characterized by loss of introns, poly A tracts, and flanking short direct repeats [15]. *TGIFLX *has 2 exons with a 96 base-pair intron [14]. TGIF2 and TGIFLX share high conservation both within the homeodomain and the C-terminus conserved region but show extensive variation outside these domains [14]. *TGIFLX *is highly conserved between primates. There is an orthologue in mice, *Tex1 *(testis-expressed homeobox 1), [16], indicating that the *TGIFLX *retrotransposition event occurred at least 80 million years ago [14]. *TGIFLX/Tex1 *expression is restricted to the adult testis [14,16]. *Tex1 *mRNA is detected in the spermatids in the seminiferous tubule and in some residual bodies, suggesting that it may play a critical role in spermatogenesis [16]. Unlike *TGIF1 *and *TGIF2*, this gene is not expressed in the brain, so it is unlikely to be involved in brain development [14].

X-linked genes are believed to evolve more rapidly than their autosomal orthologues due to their hemizygosity in males [17,18]. The mammalian sex chromosomes emerged from an ancestral pair of autosomes [19] and have received specific additions and deletions in each lineage. A large number of genes on the X-chromosome have a role in mammalian spermatogenesis [20,21]. Human *TGIFLX *maps to Xq21.3 in human [14], within a region recently added to the X-chromosome in the eutherian lineage [22] and is expressed in the testis [14]. Homeobox genes expressed in the testis such as *TGIFLX *and *ESX1L *are biased on the X-chromosome [17]. Two forms of *TGIF *genes (*Achintya *&*Vismay*) identified in *Drosophila *are crucial for spermatogenesis, suggesting that there is an ultra-conserved function for these genes in all animals [23,24].

To date, *TGIFLX *and its orthologues have only been studied in eutherian mammals. Since marsupial mammals diverged from eutherian mammals between 130 and 148 MYA [25-27], we investigated whether this gene was present in a marsupial, the tammar wallaby, *Macropus eugenii*. We characterised *TGIF1 *and *TGIF2 *orthologues in the tammar, identified their chromosomal location(s), and examined their expression throughout development and in adult tissues.

## Results

### The absence of *TGIFLX *and identification of *TGIF1 *&*TGIF2*

**Figure 1:**
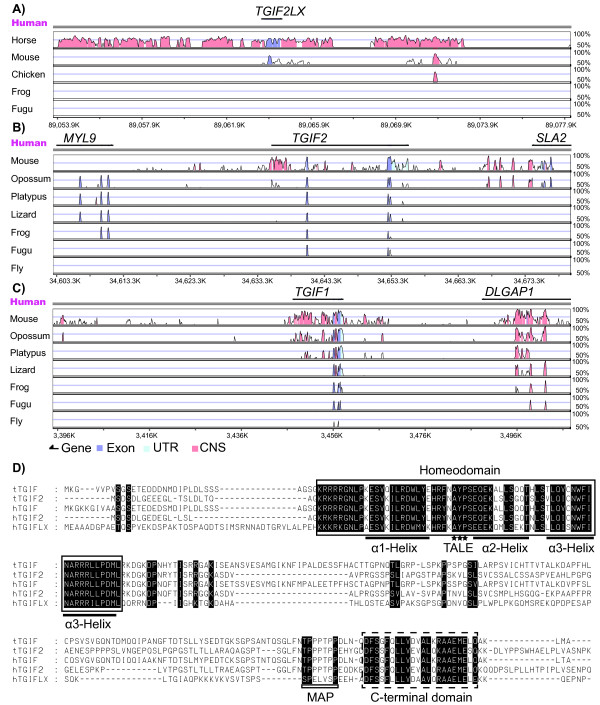
**Comparison of TGIF family**. A) *TGIFLX *orthologues are only found in eutherian mammals including human, horse and mouse, but there are no orthologues in non-mammals including chicken, frog and *Fugu *with VISTA browser. Human genomic sequence of *TGIFLX *(chrX: 89,053,736-89,078,217) compared to the horse (contig_22722: 204,584-233,963), mouse (chrX: 115,540,211-115,561,508), chicken (showing only conserved CNS in chr4: 7362172-7362401, no alignment in other region), frog (no alignment and no contigs can be shown) and *Fugu *(no alignment and no contigs can be shown). B) *TGIF2 *orthologues are present in all vertebrates, but not in invertebrates. Human genomic sequence encompassing MYL9-TGIF2-SLA2 (chr20: 34,602,792-34,680,072) compared to the mouse (chr2: 156,632,126-156,706,204), opossum (chr1: 389,963,044-390,050,278), platypus (Ultra337: 3,776,448-3,849,788), lizard (scaffold_17: 7,638,665-7,723,584), frog (scaffold_38: 522,992-673,094), *Fugu *(chrUn: 295,276,805-295,277,868) and fly (chr2R: 8,363,354-8,428,229). C) *TGIF *orthologues are present in all vertebrates and invertebrates with the conserved downstream and upstream genes in most of species. Human genomic sequence encompassing MYOM1 (partial sequence and not shown)-TGIF1-DLGAP1 (chr18: 3,395,327-3,507,935) compared to the mouse (chr17: 71,147,775-71,255,269), opossum (chr3: 269,037,689-269,144,991), platypus (Contig3116: 1-55,206), lizard (scaffold_70: 2,188,819-2,296,955), frog (scaffold_337: 1,113,822-1,225,651), *Fugu *(chrUn: 210,908,683-211,016,001) and fly (chr2R: 8,353,314-8,461,279). High similarity over a 100 bp window (showing with a peak region) is seen for exon sequence (slate blue) and untranslated regions (UTR, powder blue) or conserved non-coding sequence (CNS, red). The top double line stands for human genomic sequences. The bottom of each picture showed the human genomic sequence number. D) Amino acids alignment of human (h) TGIF family members and tammar (w) TGIF family members. Identical amino acids between the sequences are shaded black. The homeodomain is boxed and the conserved carboxyl-terminal domain (CCD) is indicated by a dashed box. The position of the three alpha helices (α1, α2, and α3) is indicated by a line below the sequence. The extra 3 residues, AYP, between helix α1 and α2 are highlighted with stars and labeled as TALE. The mitogen-activated protein (MAP) kinase phosphorylation site is indicated with a rectangular box below the sequence. The GenBank accession numbers for these proteins are: hTGIF2, (BAB16424); hTGIF1 (NM_003244) and hTGIFLX (AJ427749). Tammar TGIF1 and TGIF2 sequence have been submitted to GenBank (tTGIF: JF796112; tTGIF2: FJ775183).

Human *TGIFLX *(ENST00000283891) was used to search all available genome databases to identify *TGIFLX*-related sequences. An orthologue of *TGIFLX *was identified from the horse but its chromosomal location is unknown. Horse *TGIFLX *shared high similarity through the exons and both upstream and downstream flanking sequences. Another orthologue was identified from the mouse known as *Tex1 *which only shared partial similarity in the homeobox region. No orthologues were identified from other species including the tammar, platypus, chicken and fish (Figure 1A).

Owing to the absence of a *TGIFLX *orthologue in non-eutherian mammals, the marsupials and monotremes, another BLAST search was performed for its progenitor gene *TGIF2 *and paralogue *TGIF1 *from various species including reptiles, amphibians, fish and flies. It showed orthologues of *TGIF2 *were present in all vertebrates investigated. *TGIF2 *orthologues shared the same upstream gene *MYL9 *in most vertebrates except fish and the downstream gene *SLA2 *that was only present in therians. The *TGIF2 *orthologues were poorly conserved at the first exon (Figure 1B). No orthologue of human *TGIF2 *was identified in the fly genome. The *TGIF1 *orthologues were identified in all species examined and shared the same upstream gene *MYOM1 *(data not shown) and downstream gene *DLGAP1 *(Figure 1C).

### Characterization of tammar *TGIF1 *and *TGIF2*

Tammar *TGIF1 *and *TGIF2 *were amplified from adult tammar testis by reverse-transcriptase PCR (RT-PCR) with cross-species primers. The tammar full-length *TGIF2 *cDNA encodes a predicted protein of 252 amino acids, 15 amino acids longer than human and mouse orthologues. The predicted tammar TGIF1 protein contains 269 amino acids, 3 amino acids shorter than eutherian orthologues. Tammar partial genomic sequences from NCBI http://blast.ncbi.nlm.nih.gov/Blast.cgi were retrieved by alignment with tammar *TGIF *and used to confirm their genomic structure. Tammar *TGIF2 *has 3 exons and the open reading frame spanned all exons. The homeobox was located within the second exon. Similarly, tammar *TGIF1 *also contains 3 exons but the coding region only spanned exons 2 and 3.

Alignment of the predicted amino acid sequences of various orthologues of the TGIF family from human and tammar revealed that they were highly conserved over the homeodomain and carboxyl-terminal conserved domain (CCD) (Figure 1D). Both tammar TGIF1 and TGIF2 contained a three-amino acid loop extension (TALE) between the α1 helix and α2 helix, confirming that tammar TGIF1 and TGIF2 belong to the TALE super-family. The α3 helix was identical between human and tammar TGIF1 and TGIF2. In addition, the region containing the mitogen-activated protein (MAP) kinase phosphorylation sites (TP & PPTP) close to the carboxyl-terminal conserved domain was identical in human and tammar TGIF1/TGIF2. However, the region outside the conserved domain had a very poor similarity and identity, especially between human TGIFLX and other orthologues (Figure 1D).

**Figure 2:**
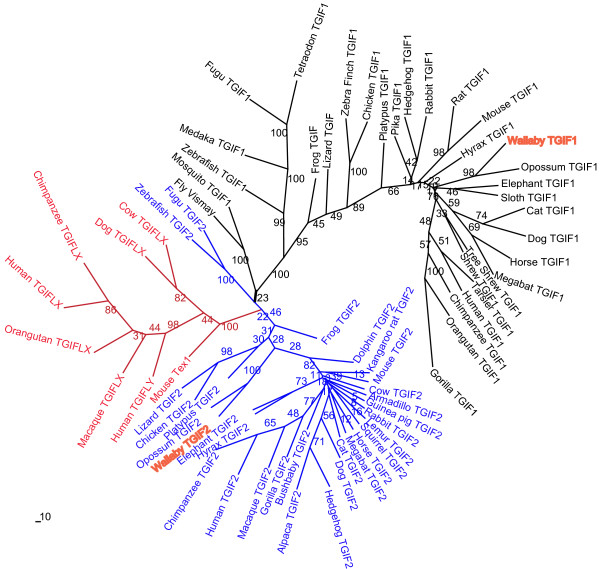
**Phylogenetic analyses with neighbour-joining showing evolutionary relationships among members of TGIF1, TGIF2 and TGIFLX/Y family**. The phylogenetic tree was constructed with PHYLIP 3.69 program. Tammar TGIF1 and TGIF2 are highlighted in bold. Numbers along branches indicate reliability of each branch with 100 replicates. TGIF1 and fly TGIF formed a branch, TGIF2 produced another branch. The TGIFLX and TGIFLY group is one of the branches of the TGIF2 cluster. GenBank (or Ensembl) accession numbers for these proteins are shown in Additional Table 1.

Full length protein sequence or near full-length protein sequence for TGIF1, TGIF2 and TGIFLX/Y homologues were retrieved from NCBI http://www.ncbi.nlm.nih.gov or Ensembl http://www.ensembl.org. There are 30 TGIF1 orthologues from various vertebrates and invertebrates, 28 TGIF2 from various vertebrates and 8 TGIFLX/Y homologues from eutherians. Phylogenetic analysis with PHYLIP program clustered these homologues into two main clusters TGIF1s and TGIF2s (Figure 2). TGIFLX/Y orthologues branched with the TGIF2s. TGIF1 from all mammals including the tammar clustered tightly, with monotremes forming the most distant branch. TGIF1 from reptiles, birds, amphibians and fish formed other branches. Invertebrates including fly and mosquito clustered as a single branch. The biggest branch of the TGIF2 cluster was the eutherian group, followed by the TGIFLX/Y cluster. Tammar and opossum TGIF2 formed a separate branch followed by monotreme, bird and reptile.

**Figure 3:**
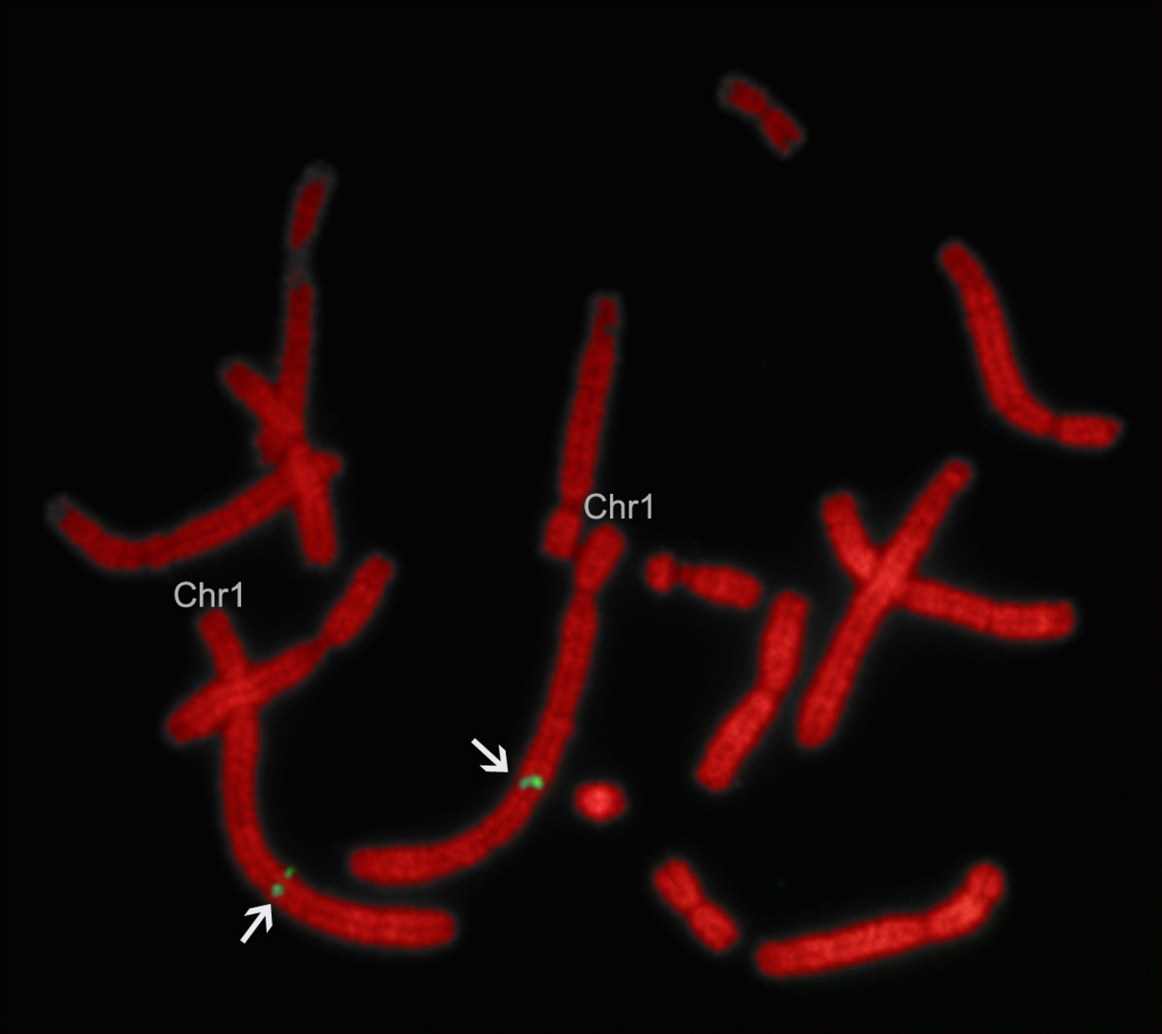
**Fluorescence *in situ *hybridization (FISH) of *TGIF2***. Hybridization signals (green) show that tammar *TGIF2 *is on the long arm of tammar metaphase chromosome 1 (Chr1; arrow).

Tammar BAC clones were identified by screening the AGI tammar BAC library with the *TGIF2 *probe. The BAC clone showing homology to tammar *TGIF2 *(67G20) was confirmed by PCR amplification. The clone was mapped to the long arm of tammar chromosome 1 by fluorescence *in situ *hybridization (FISH) (Figure 3). Only one hybridization location was detected.

### Tammar *TGIF1 *&*TGIF2 *mRNA distribution in the fetus and developing gonads

**Figure 4:**
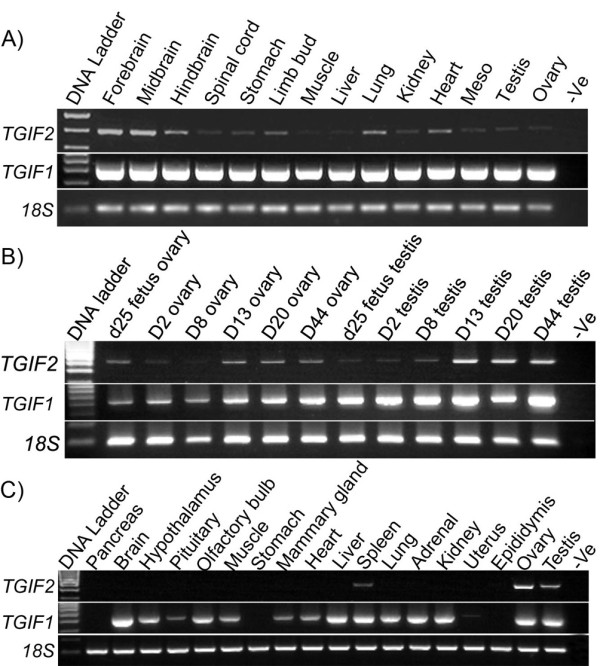
**Expression pattern of tammar *TGIF1 *and *TGIF2 *in various tissues by RT-PCR**. A) Expression of the *TGIF1 *&*TGIF2 *gene was assessed in various embryonic tissues. Both *TGIF1 *and *TGIF2 *were widely expressed in all examined tissues; B) Expression of the *TGIF1 *&*TGIF2 *during gonadal development from the bipotential gonad stage (day 25 of gestation-d25), sexual differentiation stages (days 2-3 post partum for the testis and days 7-8 for the ovary) and at later stages of development (up to day 44 post partum). Tammar *TGIF1 *and *TGIF2 *were detected at all stages; C). Expression of *TGIF1 *&*TGIF2 *was examined in various adult tissues. *TGIF1 *was broadly expressed in most tissues and *TGIF2 *was specifically expressed in the ovary, testis and spleen. PCR for 18S (100 bp) acted as a positive control and "-ve" indicates template free negative control reactions.

**Figure 5:**
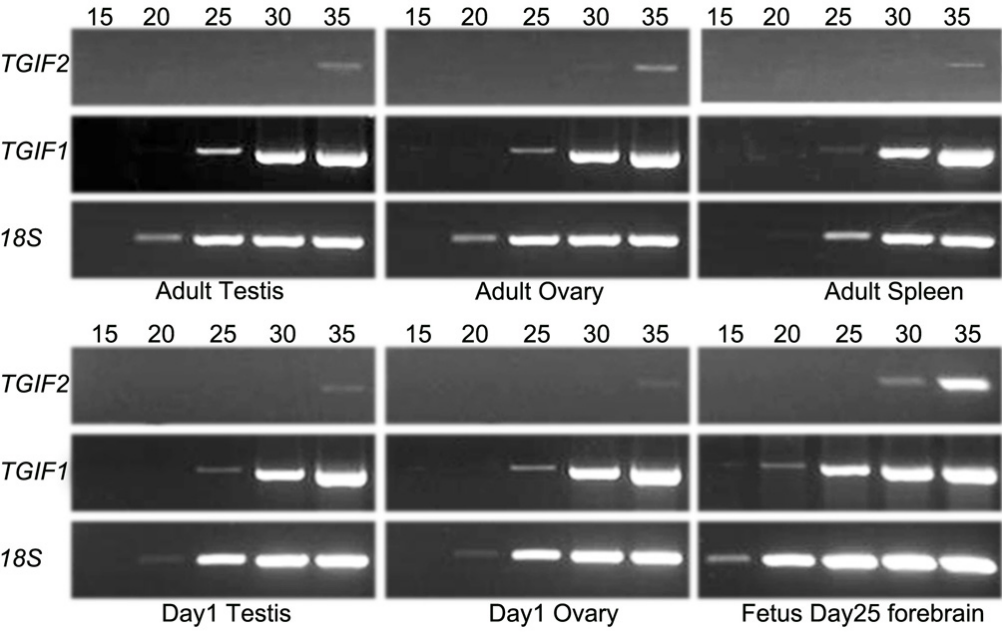
**Semi-quantitative PCR**. Identical amounts of cDNA template were utilized to identify the exponential (log) phase of the PCR reactions for TGIF1 and TGIF2 genes, while a 50 times cDNA dilution was used for amplification of the housekeeping gene 18S because of its high expression. Six tissues, adult testis, ovary and spleen as well as developing gonads and forebrain, were used for the analyses. PCRs were stopped at various cycles (15, 20, 25, 30, 35) to determine the exponential amplification phase.

RT-PCR was performed to investigate the expression pattern of *TGIF1 *&*TGIF2 *in various fetal tissues during organogenesis. Both *TGIF1 *&*TGIF2 *mRNA were detected in all the examined tissues (Figure 4A). *TGIF2 *was ubiquitously expressed during early development. Similarly, *TGIF1 *was also expressed in all examined fetal tissues. *TGIF1 *and *TGIF2 *mRNA transcripts were detected in gonads at all stages examined from the last two days of the 26.5 day gestation to day 44 *post partum *(Figure 4B and 5).

### Tammar *TGIF1 *&*TGIF2 *mRNA distribution in adult tissues

*TGIF1 *&*TGIF2 *expression was investigated in adult tissues including the adult testis and ovary. As in the human, tammar *TGIF1 *was ubiquitously expressed in most adult tissues except the pancreas, stomach and epididymis. In contrast, *TGIF2 *was only expressed in the adult gonads and was absent in all other tissues except the spleen (Figure 4C and 5). To further confirm our results, we performed semi-quantitative PCR (Figure 5). This further demonstrated that *TGIF1 *has a broader expression pattern than *TGIF2*.

### Tammar *TGIF2 *mRNA and protein distribution during spermatogenesis

**Figure 6:**
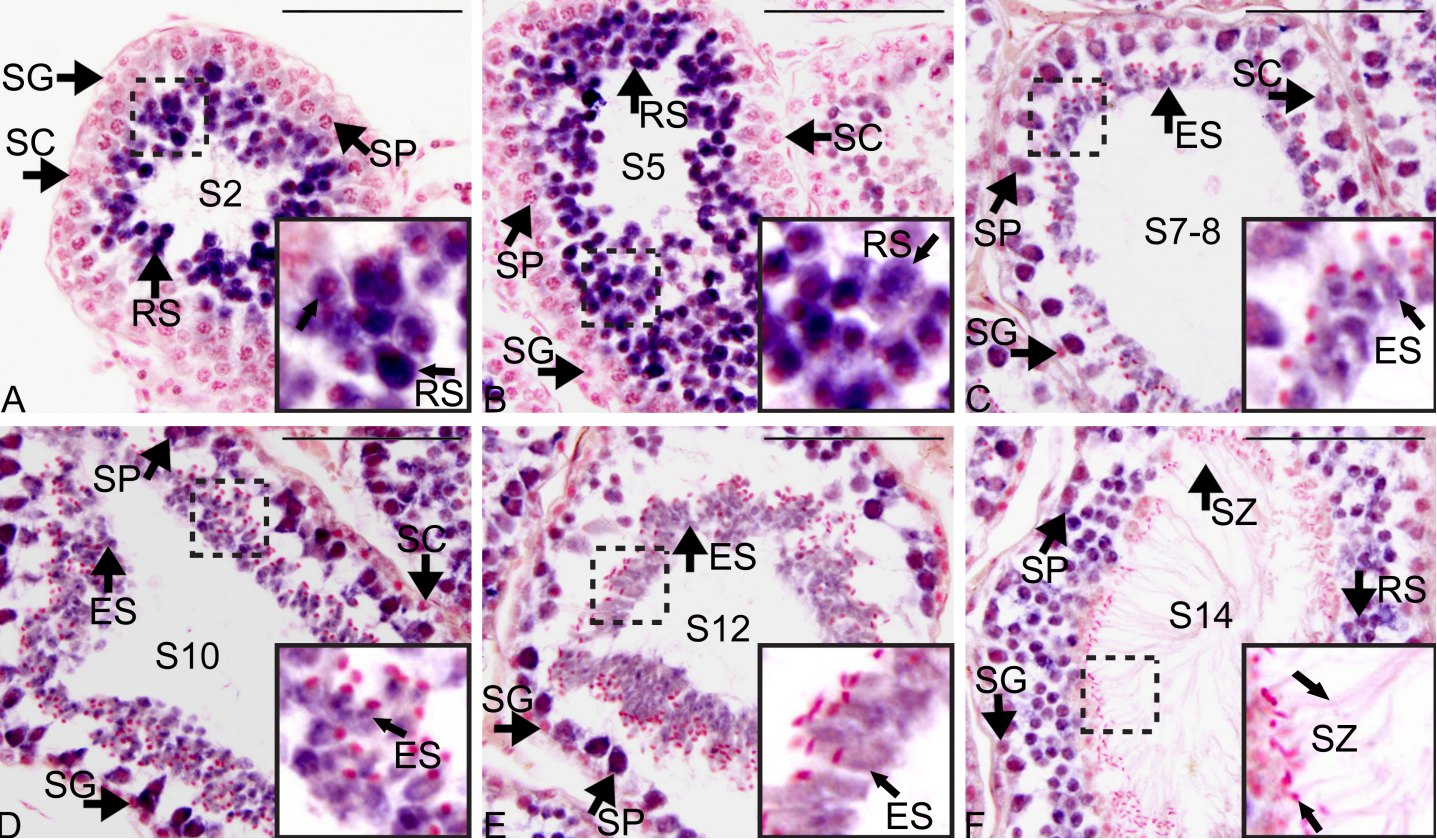
**mRNA *in situ *hybridization of *TGIF2 *during spermatogenesis in tammar adult testis**. Each panel shows a seminiferous tubule at different stages of the spermatogenic cycle indicated by the steps 1 to 14 (S1 to S14) [28]. All images shown were hybridized with antisense probe (purple blue) and counterstained with nuclear fast red. Images boxed on the right bottom are the high resolution pictures from the corresponding part highlighted with dashed boxes. *TGIF2 *mRNA was detected in the cytoplasm of early round spermatids (A, step 2) and round spermatids before transforming to elongated spermatids (B, step 5). Staining was also observed in the cytoplasm of elongated spermatids and spermatocytes (C-E, step7-12). Image F indicates that there was no staining on the mature spermatozoa with arrows showing the sperm head (bottom arrow) and sperm tail (top arrow) (step 14). SG, spermatogonia; SP, spermatocytes; RS, round spermatids; ES, Elongating/elongated spermatids; SZ, spermatozoa; SC, Sertoli cells. Scale bars = 100 μm.

In the adult testis, using *in situ *hybridization *TGIF2 *mRNA was detected in the germ cells, predominantly in a layer around the seminiferous cord lumen (Figure 6). No mRNA staining was detectable in the Sertoli cells or interstitial cells. There was strong *TGIF2 *mRNA staining in round spermatids when they were just newly formed (step 2, [28], Figure 6A) and before transforming into elongated spermatids (step 5, Figure 6B). After round spermatids started to elongate, staining decreased moderately but was still detectable in the cytoplasm of elongating/elongated spermatids and spermatocytes (step 7-8, Figure 6C; step 10, Figure 6D; step 12, Figure 6E). No staining was detected when elongated spermatids transformed into mature spermatozoa and were released into lumen (step 14, Figure 6F).

**Figure 7:**
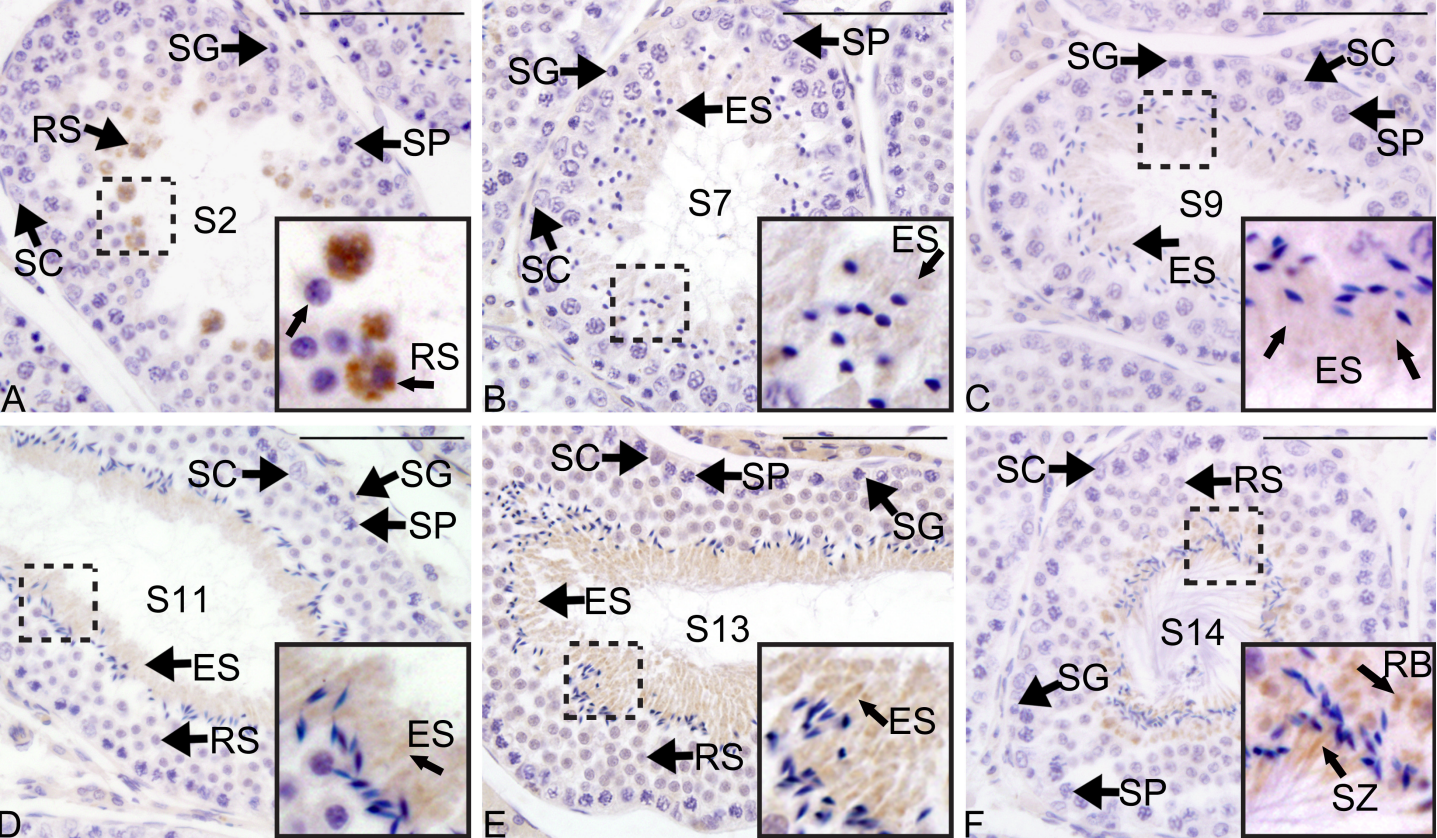
**Immunohistochemistry of TGIF2 protein during spermatogenesis in the tammar adult testis**. Each panel shows a seminiferous tubule at different stages of the spermatogenic cycle. Sections were counterstained with haematoxylin (blue). TGIF2 protein (brown staining indicated with arrow) is strongly detected in the cytoplasm of early round spermatids (A, step 2) and was much weaker or undetectable in the later stage round spermatids (B, step 7). As round spermatids transformed into mature spermatozoa, staining decreased dramatically and was almost undetectable in the elongated spermatids (C-E, step 9-13). F shows immunostaining in the residual body (top arrow) and mid-piece (bottom arrow) of the sperm tail (step 14). Images boxed on the right bottom are the high resolution pictures from the corresponding part highlighted with dashed box. SG, spermatogonia; SP, spermatocytes; RS, round spermatids; ES, Elongating/elongated spermatids; SZ, spermatozoa; SC, Sertoli cells; PC, peritubular myoid cells; RB, residual bodies. Scale bars = 100 μm. Steps of spermatogenesis defined in [28].

TGIF2 protein was also restricted to the germ cells during spermatogenesis. When round spermatids were newly formed, TGIF2 protein was present and there was strong staining in the cytoplasm of spermatids (step 2, Figure 7A). After that, immunostaining decreased significantly to an almost undetectable level (step 7-11, Figure 7B-D). At late stages of spermatid transformation, TGIF2 was detected to the cytoplasm of germ cells surrounding the lumen (step 13, Figure 7E). When the sperm tail was fully formed and released to the lumen, TGIF2 protein was localized in the residual body and the mid-piece of sperm tail (Figure 7F).

### Tammar *TGIF2 *mRNA distribution during folliculogenesis

**Figure 8:**
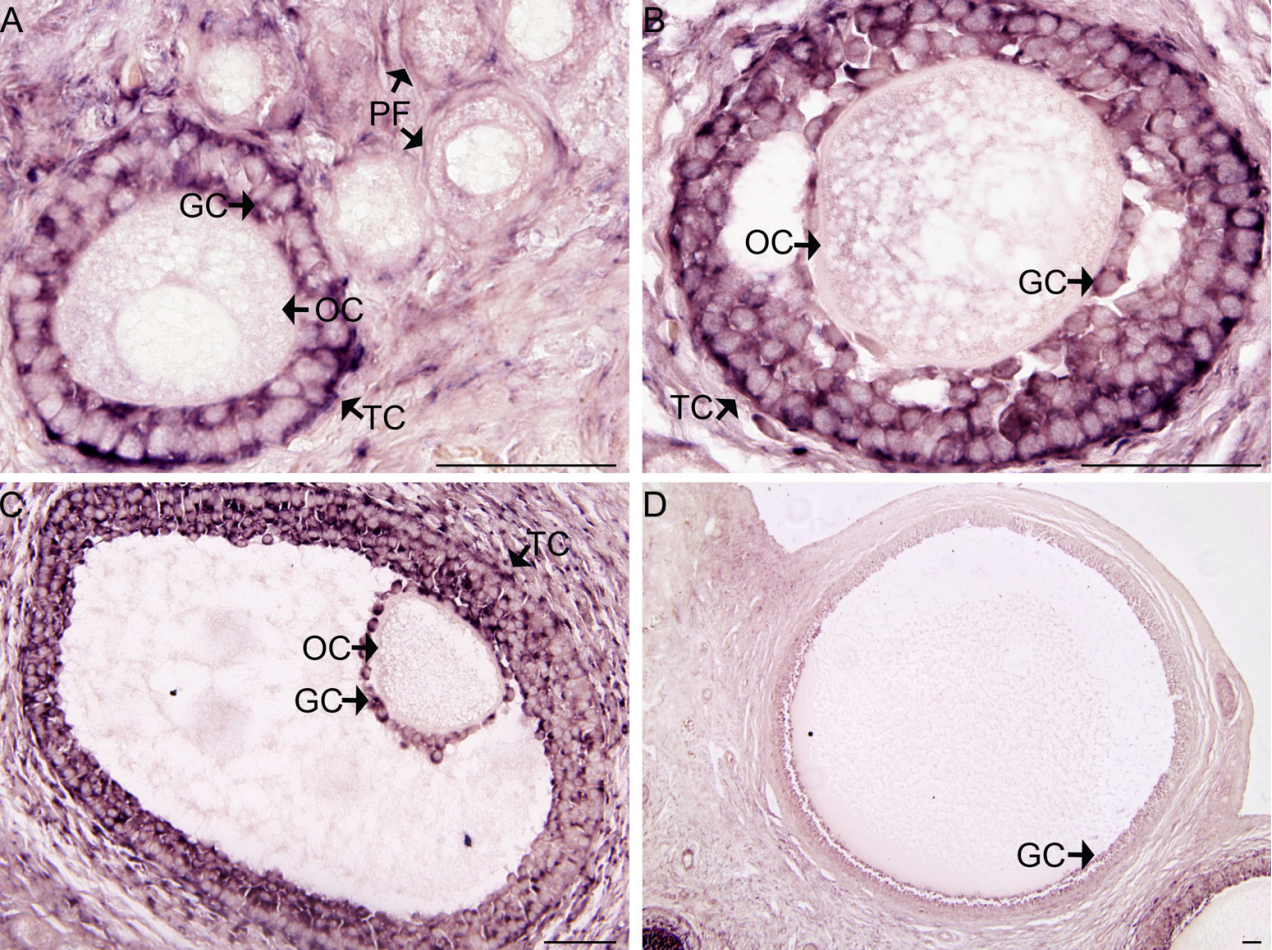
**mRNA *in situ *hybridization of *TGIF2 *in tammar adult ovary**. Sections were hybridized with antisense probe without counterstaining. *TGIF2 *mRNA (purple blue) was predominantly detected in the granulosa cells and theca cells of the secondary follicles (A & B) and tertiary follicles (C). No staining was detected in primary follicles (PF) (A) and *TGIF2 *mRNA was almost invisible in Graafian follicles (GF) (D). There was no staining detected in the oocytes. Signal was indicated with arrow. PF, primary follicle; GC, granulosa cell; TC, theca cell; OC, oocyte. Scale bars = 50 μm.

In the adult ovary *TGIF2 *mRNA was detected in the granulosa cells and theca cells of the secondary follicles and tertiary follicles (Figure 8A-C). There was no mRNA detected in the primary follicles (Figure 8A) and *TGIF2 *was almost undetectable in the Graafian follicle (Figure 8D). There was no staining seen in the oocyte.

## Discussion

### Conservation of the TGIF family in vertebrates

*TGIF1 *and *TGIF2 *share the highest degree of homology in the TGIF family. The characterisation of *TGIF1 *and *TGIF2 *established that both genes have 3 exons in all species except the orthologue of *TGIF1 *in *Drosophila*. The conservation of *TGIF1 *and *TGIF2 *throughout the exons, the UTR and flanking regions suggests the functional importance of these genes during evolution. The X-linked *TGIFLX *is specific to the eutherian lineage and arose after they diverged from marsupials and monotremes around 148 and 166 million years ago respectively.

**Figure 9:**
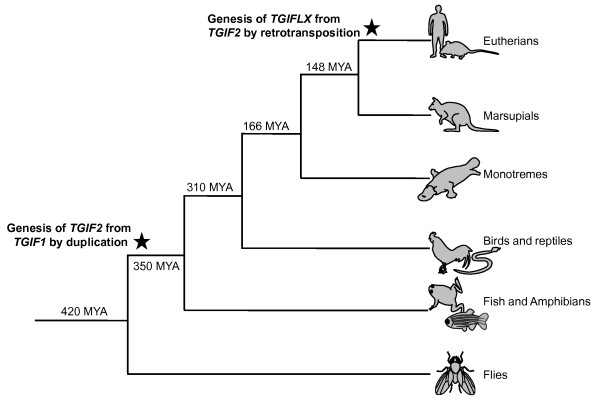
**Proposed model of *TGIFs *evolution**. *TGIF2 *emerged from *TGIF1 *by gene duplication about 420MYA while *TGIFLX *was generated from *TGIF2 *by a retrotransposon after eutherians diverged from marsupials about 148MYA.

The entire tammar TGIF2 protein shared 71% amino acid identity with human TGIF2 and only 33% similarity with human TGIFLX. The high degree of conservation over the homeodomain and TALE regions suggests that TGIF1 and TGIF2 interactions via the homeodomain may be similar in marsupial as in human and mouse [4,5]. The third helix within the homeodomain, critical for DNA binding, is the most conserved between eutherian and marsupial orthologues, suggesting they are likely to bind to the same DNA motif as identified in the mouse [4,7]. Phylogenetic analyses showed that the *TGIFLX *groups tightly with the TGIF2 branch, supporting the suggestion that *TGIFLX *originated from a *TGIF2 *retrotransposition event [14,17] (Figure 9).

### Both *TGIF1 *&*TGIF2 *are important for embryonic and gonadal development

Marsupial *TGIF1 *and *TGIF2 *mRNA was expressed in all embryonic tissues examined, as in mice [11,13]. Tammar *TGIF2 *mRNA was abundant in embryonic brain, consistent with the high level seen in the murine embryonic nervous system [13]. Tammar *TGIF1 *was observed in all examined tissues, again consistent with previous studies in mice, and there was abundant mRNA expression in the brain [10,11]. In contrast, mouse *Tgif1 *mRNA transcripts were restricted in the brain from E15 indicating that *Tgif1 *may be involved in cerebellum development and maturation [11]. The overlapping expression of these two genes and the phenotype of the *Tgif1 *knockout mouse suggest that *Tgif2 *can compensate for the loss of *Tgif1 *during embryogenesis [10]. The ubiquitous expression of *TGIF1 *and *TGIF2 *in the tammar similarly suggests that both *TGIF1 *and *TGIF2 *may be functionally redundantly during marsupial embryogenesis.

### Differential function of tammar *TGIF1 *and *TGIF2 *in adult tissues

Tammar *TGIF1 *was broadly expressed in adult tissues including the testis, in similar locations to those described for the human and mouse [3,11]. Mouse Tgif1 is found in the nuclei of peripheral regions of tubules such as spermatogonia and primary spermatocytes in the adult testis [23]. Murine *Tgif2 *mRNA transcripts are present and functional as transcriptional repressors in adult and embryonic tissues and have similar expression patterns [9]. Similarly, *TGIF2 *is ubiquitously expressed in human tissues [12]. In contrast, tammar *TGIF2 *was exclusively expressed in the adult testis, ovary and spleen. The expression of tammar *TGIF2 *in adult gonadal tissues is instead similar to that of human *TGIFLX *and mouse *Tex1 *that are expressed only in adult testis and are thought to be involved in spermatogenesis [14,16,17]. Marsupial *TGIF2 *was strongly expressed in round spermatids but appeared to be down-regulated as they transformed into elongated spermatids, identical to the *Tex1 *expression pattern in mice [16]. Tammar TGIF2 protein was strongly detected in early round spermatids and in mature spermatids, and also detected in the residual bodies, in line with *Tex1 *mRNA distribution in mice [16]. Since *TGIFLX *is undetectable in some humans with abnormal spermatogenesis, *TGIFLX *is likely to have a function in male reproduction and development [29]. Therefore, the expression pattern of marsupial TGIF2 and its specific localisation in the germ cells during spermatogenesis suggests that TGIF2 has developed a specialised function in marsupial germ cells.

The mRNA of tammar *TGIF2 *was examined in the adult ovary and was detected primarily in the granulosa cells in developing ovarian follicles, predominantly in secondary and tertiary follicles, suggesting that *TGIF2 *may also function in folliculogenesis and the development of oocyte. This is the first examination of *TGIF2 *in ovaries of any mammal. *TGIF2 *was also detected in the thecal cells, known to be critical for follicular function [30,31].

### Evolution of the TGIF family from fly to human: specialised function in sex and reproduction?

The mammalian X-chromosome is known to contain a disproportionate number of genes involved in testis and brain function [19,32]. This is thought to have evolved due to direct selection of male advantage genes on the hemizygous × [17]. Whether X-linked genes are more likely to evolve testis functions or testicular genes are more likely to be relocated to the × remains to be tested. The marsupial X-chromosome is the smallest × in mammals and is most similar to the X-chromosome of the therian ancestor [32,33]. In the eutherian lineage the × has been expanded, mainly by the addition of two large autosomal blocks from the therian ancestor. These two large additions are autosomal in the marsupial and non-mammalian vertebrate lineages [33-36]. *TGIFLX *maps to the conserved region of the X-chromosome, present in all therian mammals, but was absent on the marsupial X-chromosome. Eutherian *TGIFLX *was not part of the two large autosomal additions but arose via retrotransposition, presumably from TGIF2 [14]. Once on the eutherian X-chromosome it was exposed to positive selection that favours the evolution of male reproductive advantage genes [17,21], possibly leading to its specialisation in the adult testis. The accelerated rate of *TGIFLX *evolution therefore appears to be due its location on the × and its potentially redundant function with its progenitor *TGIF2 *[17,37]. Despite the autosomal location of tammar *TGIF2*, it too has a specialised role in the adult marsupial in spermatogenesis and possibly folliculogenesis. The conflicting expression profiles of *TGIF2 *in adult marsupials and eutherians make it impossible to determine if the ancestral *TGIF2 *orthologue was a broadly expressed gene in the adult or was gonad-specific. There is good evidence that the × selectively retains translocated testis-specific genes that may confer a male advantage [21,32,33]. Thus the ancestral *TGIF2 *may have already had a function in mammalian spermatogenesis before a translocated copy moved to the × in eutherians. Due to the new *TGIFX *orthologue now fulfilling the testicular function, autosomal *TGIF2 *was able to diversify and take on broader functions in the adult. This suggestion is supported by the similar degree of functional specialisation in reproduction from the autosomal copy of *TGIF2 *in marsupials, demonstrating that a location on the sex chromosome is not the only the driving force behind the rapid specialisation of this gene in spermatogenesis.

## Conclusions

This study demonstrates that tammar *TGIF1 *had a similar expression pattern to that of eutherians [11], but *TGIF2 *has a restricted expression pattern, very similar to that of eutherian *TGIFLX*/*Tex1*. Since *TGIFLX *and its orthologues are only present in the eutherian lineage, the retrotransposition event resulting in its creation must have occurred after the marsupial-eutherian divergence between 130 and 148MYA [25] but before ~80 MYA when the primate-rodent lineages split [14,17]. The germ cell specific expression of marsupial *TGIF2 *in adult testes suggests it may have a role in spermatogenesis, similar to that of *TGIFLX*/*Tex1 *in eutherian mammals and of the *TGIF *genes in *Drosophila*. *TGIF2 *expression was shown for the first time in follicles and oocytes so it is likely that it also has a role in folliculogenesis. Together these data suggest that there has been an ultra-conserved function for the TGIF family in fertility and that *TGIF2 *already had specialised role in the adult, in spermatogenesis and folliculogenesis long before its retrotransposition in eutherian mammals.

## Methods

### Gene cloning and structure

Both *TGIF1 *and *TGIF2 *were initially cloned by RT-PCR using cross-species primers (MeTGIFF1 and MeTGIFR1 for *TGIF1*, TF1 and TR1 for *TGIF2*) based on conserved regions in the human, mouse and opossum genomes. The resulting 554 bp PCR product (*TGIF2*) was sequenced and then used to design tammar specific primers for 3' and 5' RACE to clone the full *TGIF2 *mRNA transcript [GenBank Accession No. FJ775183]. Sequencing primers are listed in Table 1.

**Table 1:** Primers designed for analysis of *TGIF1 *&*TGIF2 *expression by RT-PCR

Primers	Sequence (5'→3')	Function
meTGIFF1	CGAGACTGGCTCTATGAA	RT-PCR
meTGIFR1	AGATTTACCCGTGTCCTC	RT-PCR
TF1	TGAAGAT(C/T)CTCCGAGACTGG	Cross species cloning & RT-PCR
TR1	CCACCAGCAG(G/C/T)TGGAAGC	Cross species cloning & RT-PCR
TF2	ATGGCAAGGACCCTAACCG	3' RACE and RT-PCR
TR2	TGGAAGAAGCCGCCGTCGTG	5' RACE
SMART IV	AAGCAGTGGTATCAACGCAGAGTGGCCATTACGGCCGGG	5' RACE
CDS III	ATTCTAGAGGCCGAGGCGGCCGACATG-d(T)_30_N_-1_N (N = A, G, C, or T; N-1 = A, G, or C)	3' RACE
5'PCR primer	AAGCAGTGGTATCAACGCAGAGT	5' RACE
18S F	GATCCATTGGAGGGCAAGTCT	RT-PCR
18S R	CCAAGATCCAACTACGAGCTTTTT	RT-PCR

F denotes forward primers, R denotes reverse primers. All primers are shown in the 5'→3' direction; CDSIII, Smart IV and 50 PCR were synthesized by SIGMA (Genosys) according to the manual of the SMART cDNA library construction kit from Clontech.

cDNA was reverse-transcribed from total RNA isolated from an adult tammar wallaby testis, using the SMART cDNA library construction kit (Clontech, Mountain View, California, USA). 5' RACE was performed using primer 5' PCR and primer TR1. Due to an incomplete coding sequence at the 5' end, we designed a new primer TR2 as a nested primer and repeated 5' RACE. 3' RACE was performed using primer TF1 and CDS III, nested PCR was performed using the TF2 and CDS III primers. PCR cycling conditions were: 35 cycles of 30s, 95°C; 60s, 55°C; 120s, 72°C, in a 25 μl reaction with GoTaq Green Master Mix (Promega, Wisconsin, USA; Primers listed in Table 1).

Genomic sequence of tammar *TGIF1 *and *TGIF2 *were retrieved from the tammar wallaby genome trace archives http://www.ncbi.nlm.nih.gov/. These reads were aligned with the full length cDNA sequence to determine the gene structure of *TGIF2*. This confirmed the number and the length of exons.

### Genomic sequence analysis and phylogenetic tree

Human genomic sequences for *TGIFLX*, *TGIF2 *and *TGIF1 *were obtained from the UCSC genome browser http://genome.ucsc.edu/. The conserved block of genes surrounding these loci were identified in amniote species using the VISTA genome browser program http://pipeline.lbl.gov/cgi-bin/gateway2. Blast was then performed in mouse, opossum, platypus, lizard, frog, fugu and fly. Genomic sequence from these orthologous blocks was also obtained from UCSC and aligned using the LAGAN algorithm available on the mVISTA website with default parameters http://genome.lbl.gov/vista/mvista/submit.shtml except that TGIFLX orthologues were aligned with PROLAGAN algorithm. In an attempt to identify any TGIFX orthologues in marsupials or platypus we performed a low stringency BLAST using the individual human TGIFX exons as well as a protein BLAT. In each case no homology was found.

Searches for homologues of *TGIF1*, *TGIF2 *and *TGIFLX/Y *were performed at NCBI http://www.ncbi.nlm.nih.gov and Ensembl http://www.ensembl.org and orthologues were found in eutherians, marsupials, monotreme (platypus), birds and reptiles, fish and invertebrates (fruit fly and mosquito) (Additional file 1, Table S1). Phylogenetic analysis was performed with PHYLIP 3.69 program with default parameters (University of Washington) using neighbour-joining analysis with 100 replicates, and viewed with TREE-view 1.66 http://taxonomy.zoology.gla.ac.uk/rod/treeview.html.

### RT-PCR

RT-PCR was used to check the mRNA expression pattern of *TGIF1 *and *TGIF2 *in embryonic tissues (fetal stage at day 25 in 26.5 days gestation), developing gonads (from fetal stage before birth to pouch young stage of day 44 after birth) and adult tissues (Pancreas, brain, hypothalamus, pituitary, olfactory bulb, muscle, stomach, heart, liver, spleen, lung, adrenal, kidney, uterus, epididymis, ovary and testis). Total RNA was isolated using RNAwiz (Ambion Inc., Austin, Texas, USA) according to the manufacturer's instructions. The quality and quantity of total RNA was verified by gel electrophoresis and optical density reading with a Nanodrop (ND-1000 Spectrophotometer, Wilmington, USA). 2 μg of total RNA was DNase-treated with *DNase *I (Ambion Inc., Austin, Texas, USA) for 30 minutes. 1 μg of DNased RNA was reverse-transcribed with oligodT primer using the SuperScript III kit (Invitrogen, California, USA).

PCR was performed in a 25 μl reaction with GoTaq Green Master Mix, primers (meTGIF1F and meTGIF1R for *TGIF1*, TF2 and TR1 for *TGIF2*) and first-strand cDNA products. Amplification conditions were: 95°C 30s; 49.2°C 30s; 72°C, 30s for 35 cycles (*TGIF1*) and 95°C 30s; 57°C 30s; 72°C, 30s for 35 cycles (*TGIF2*), or 25 cycles (18S). Samples were analyzed on a 2% agarose gel. Furthermore, adult tissues (testis, ovary and spleen), developing gonads (testis and ovary at day 1 after birth) and embryonic tissue (forebrain at day 25 of gestation) expressing both *TGIF1 *and *TGIF2 *were selected to validate that our PCR results were within the exponential amplification phase of the PCR [38]. Briefly, same amount of cDNA template was used for *TGIF1 *and *TGIF2 *amplifications and 50 times dilution of cDNA template was used for 18S amplification. PCRs were stopped after a variable number of cycles (15, 20, 25, 30 and 35) to determine the exponential phase. For the *TGIF2 *primers, 35 cycles was within the exponential phase; for *TGIF1 *primers, 35 cycles almost reached the plateau phase, but was still within the exponential phase; for *18S *primers, amplification had plateaued by 30 cycles, but was in the exponential phase after 25 cycles.

### mRNA *in situ *hybridization

Antisense and sense RNA probes were prepared separately from a region including *TGIF2 *domain (~500 bp, spanning nucleotides 178-732) of the tammar wallaby transcript using SP6 and T7 polymerase respectively and labeled with digoxigenin-UTP (Roche, Castle Hill, NSW, Australia, Cat#10999644001). Tissues were fixed in 4% paraformaldehyde overnight at 4°C rinsed several times in 1X PBS (140 mM NaCl, 2.7 mM KCl, 10 mM Na_2_HPO4, 1.8 mM KH_2_PO4, pH = 7.4), embedded in paraffin, sectioned at 8 um and placed onto polysine coated slides (Menzel-Gläser, Germany). After dewax and rehydration, the sections were washed several times with 1X PBS, 0.1 M Glycine, 0.1% Triton X-100 and freshly made triethanolamine (TEA) buffer (100 mM triethanolamine, 0.25% (v/v) acetic anhydride, pH = 8.0), and then were immediately pre-hybridized in prehybridisation buffer containing 10% Dextran sulfate, 1× Denhardt's solution, 4× SSC, 50% Deionized formamide, 2 mM EDTA and 500 μg/ml herring sperm DNA for 1 hours at 37°C. Hybridization was performed on each section with 50-100 μl hybridization buffer containing 100 ng/ml of DIG-labeled RNA probe for 16-18 hours at 42°C. Hybridization signals were detected immunologically with alkaline phosphatase-conjugated anti-DIG antibody (Roche, Castle Hill, NSW, Australia, Cat# 11093274910) and visualized with NBT/BCIP (Roche, Castle Hill, NSW, Australia, Cat# 11681451001) according to the manufacturer's instructions. Tissues were counterstained with 0.1% nuclear Fast Red (Aldrich Chemical Corp., Milwaukee, WI, USA).

### Immunohistochemistry

Fresh tissues were collected and fixed in 4% paraformaldehyde and treated as described above. Then tissue sections were dewaxed in histolene (Grale Scientific, VIC, Australia) and rehydrated in a series of ethanol/water solutions. Antigen retrieval was performed in boiling sodium citrate buffer (10 mM sodium citrate, 0.05% Tween 20, pH 6.0) for 20 minutes, sections were then treated with 5% hydrogen peroxide in methanol for 20 minutes. The primary antibody (TGIF2 purified MaxPab rabbit polyclonal antibody, Cat# H00060436-D01P, Abnova, USA) was applied to tammar adult gonad tissue sections at 1:150 dilutions and incubated at 4°C overnight. Signal was amplified using the streptavidin/HRP kit (DAKO, Australia, Cat# P0397), visualized with DAB (DAKO, Australia, Cat# 1967), and counterstained with hematoxylin.

### BAC library screening and chromosomal mapping

To establish the chromosomal localization of *TGIF2 *in the tammar, a BAC library was screened with a *TGIF2 *554 bp PCR product as a probe. Membranes were pre-hybridized for 2 hours in Church buffer (0.25 M NaHPO4, 1 mM EDTA, 1% BSA and 7% SDS). 25 ng of probe PCR product was labeled with the Amersham Megaprime DNA Labeling System (GE Healthcare, Rydalmere, NSW, Australia, Cat# RPN1606) with [α-^32^P] dCTP (10 mCi/ml, PerkinElmer, Melbourne, VIC, Australia) following the manufacturer's instructions. Hybridization was performed at 65°C overnight. Filters were washed with 2XSSC/0.1%SDS, 1XSSC/0.1%SDS, and 0.1XSSC/0.1%SDS for 10 minutes each at 65°C and autoradiographed at -70°C for 2-5 days [39]. BACs were identified based on a duplicate spot pattern according to the Arizona Genomics Institute protocol http://www2.genome.arizona.edu/research/protocols.

Chromosome preparations were made from pouch young testis cells according to standard methods with minor modifications [40]. Chromosome fluorescence *in situ *hybridization (FISH) was performed as previously described with minor modifications [41]. The BAC genomic DNA was labeled with dUTP-digoxygenin (DIG) by nick translation at 14°C for one hour and pre-blocked with tammar wallaby Cot-1 DNA prior to hybridization. The probe was hybridized to tammar metaphase chromosome spreads at 37°C overnight. Hybridization was detected using mouse anti-DIG-FITC antibody (Roche, Castle Hill, NSW, Australia, Cat#. 11207741910; 1/200). After hybridization, the chromosome preparations were stained with DAPI (4, 6-diamidino-2-phenylindole) (Vector lab. Inc., Burlgamine, CA, USA, Cat# H-1200) to visualize the chromosomes. Images were taken using a Zeiss microscope.

## Authors' contributions

All authors participated in the design of the study and collected the tissue samples. YH performed all the experiments, HY participated in FISH and ISH, AJP also participated in FISH. All authors analyzed the results. YH and HY drafted the manuscript. All authors read, modified and approved the final manuscript.

## Supplementary Material

Additional file 1 **Table S1**. Homologues of TGIF family from various species. Click here for file
