# Supplementary material for: Differential roles of TGIF family genes in mammalian reproduction
Source: BMC Dev Biol. 2011 Sep 29;11:58. doi: 10.1186/1471-213X-11-58 (PMC3204290; doi:10.1186/1471-213X-11-58)
Supplement: Additional file 1 — Table S1. Homologues of TGIF family from various species. [file 1471-213X-11-58-S1.DOC]

**Table 1 Homologues of TGIF family from various species**

| **Group** | | **Species** | **Accession number** | **Group** | | **Species** | **Accession number** |
| --- | --- | --- | --- | --- | --- | --- | --- |
| **TGIF** | Eutherian mammals | Cat | ENSFCAP00000010607 | **TGIF2** | Eutherian mammals | Cat | ENSFCAP00000012018 |
|  |  | Chimpanzee | ENSPTRP00000016744 |  |  | Chimpanzee | ENSPTRP00000023101 |
|  |  | Dog | ENSCAFP00000033761 |  |  | Cow | ENSBTAP00000006955 |
|  |  | Elephant | ENSLAFP00000001968 |  |  | Dog | ENSCAFP00000012560 |
|  |  | Gorilla | ENSGGOP00000012384 |  |  | Dolphin | ENSTTRP00000005796 |
|  |  | Hedgehog | ENSEEUP00000004420 |  |  | Elephant | ENSLAFP00000012374 |
|  |  | Horse | ENSECAP00000008963 |  |  | Gorilla | ENSGGOP00000011490 |
|  |  | Human | NM_003244 |  |  | Guinea Pig | ENSCPOP00000012299 |
|  |  | Hyrax | ENSPCAP00000003153 |  |  | Hedgehog | ENSEEUP00000000784 |
|  |  | Megabat | ENSPVAP00000006819 |  |  | Horse | ENSECAP00000008225 |
|  |  | Mouse | NM_009372 |  |  | Human | BAB16424 |
|  |  | Orangutan | ENSPPYP00000010134 |  |  | Hyrax | ENSPCAP00000010860 |
|  |  | Pika | ENSOPRP00000000881 |  |  | Kangaroo rat | ENSDORP00000007934 |
|  |  | Rabbit | ENSOCUP00000011832 |  |  | Lemur | ENSMICP00000000333 |
|  |  | Rat | NP_001015020 |  |  | Macaque | ENSMMUP00000010733 |
|  |  | Shrew | ENSSARP00000002524 |  |  | Megabat | ENSPVAP00000010133 |
|  |  | Sloth | ENSSARP00000002524 |  |  | Mouse | NM_173396 |
|  |  | Tarsier | ENSTSYP00000008236 |  |  | Rabbit | ENSOCUP00000000556 |
|  |  | Tree Shrew | ENSTBEP00000007665 |  |  | Squirrel | ENSSTOP00000001539 |
|  | Marsupials | Opossum | ENSMODP00000026815 |  | Marsupials | Opossum | ENSMODP00000001678 |
|  |  | Tammar | JF796112 |  |  | Tammar | FJ775183 |
|  | Monotreme | Platypus | ENSOANP00000022323 |  | Monotreme | Platypus | ENSOANP00000007840 |
|  | Birds & reptiles | Chicken | NP_990710 |  | Birds & reptiles | Chicken | ENSGALT00000030938 |
|  |  | Frog | NP_989382 |  |  | Frog | ENSXETT00000037691 |
|  |  | Lizard | ENSACAP00000009371 |  |  | Lizard | ENSACAP00000009534 |
|  |  | Zebra Finch | ENSTGUP00000010338 |  | FISH | Fugu | scaffold 318 |
|  | Fish | Fugu | ENSTRUP00000045769 |  |  | Zebrafish | ENSDARP00000112074 |
|  |  | Medaka | ENSORLP00000012963 | **TGIFLX/Y** | Eutherian mammals | Chimpanzee | ENSPTRP00000037983 |
|  |  | Tetradon | ENSTNIP00000015746 |  |  | Cow | ENSBTAP00000049288 |
|  |  | Zebrafish | NP_955861 |  |  | Dog | ENSCAFP00000025715 |
|  | Invertebrates | Mosquito | XP_308755 |  |  | Human X | AJ427749 |
|  |  | Fruit fly | NP_523714 |  |  | Human Y | ENSG00000176679 |
| TGIF2 | Eutherian mammals | Alpaca | ENSVPAP00000003747 |  |  | Macaque | ENSMMUP00000040939 |
|  |  | Armadillo | ENSDNOP00000007894 |  |  | Mouse | NM_153109 |
|  |  | Bushbaby | ENSOGAP00000000794 |  |  | Orangutan | ENSPPYP00000022975 |
